# Time Course of Antispike Antibody Titer after Administration of BNT162b2 mRNA COVID-19 Vaccine in a Patient with Rheumatoid Arthritis on Methotrexate

**DOI:** 10.1155/2023/4525249

**Published:** 2023-04-19

**Authors:** Satoshi Shinohara, Yasuhiro Hirose

**Affiliations:** ^1^Tochigi Rheumatology Clinic, Ekimaedori 1-1-9, Utsunomiya, Tochigi 321-0964, Japan; ^2^Moka Neurosurgical Clinic, Ohyahonmachi 3-20, Moka, Tochigi 321-4333, Japan

## Abstract

Methotrexate, an anchor drug for rheumatoid arthritis, hinders the immunogenicity of mRNA COVID-19 vaccines. Therefore, an optimal vaccine strategy for patients with rheumatoid arthritis receiving methotrexate is vital. We monitored antispike antibody titers after BNT162b2 mRNA COVID-19 vaccination in seven healthcare workers and one methotrexate-treated rheumatoid arthritis patient. The antispike antibody titers of healthcare workers significantly increased immediately after primary vaccination and then continued to decrease, whereas those of the rheumatoid arthritis patient were significantly lower immediately after primary vaccination and then increased. The titers in all participants dramatically increased 1-month postbooster. These changes over time may suggest that in the methotrexate-treated rheumatoid arthritis patient, the generation of short-lived plasma cells was strongly suppressed; in contrast, the generation of long-lived plasma cells and memory B cells was intact. For methotrexate-treated rheumatoid arthritis patients, it is important to complete the primary and booster vaccination series to ensure sufficient immunity against COVID-19.

## 1. Introduction

Patients with rheumatoid arthritis (RA) experienced a higher incidence of COVID-19 during the SARS-CoV-2 pandemic [1]. Methotrexate (MTX), an anchor drug for RA, hampers the immunogenicity of the mRNA COVID-19 vaccine [2–10]. Patients on therapeutic immunosuppressants for immune-mediated inflammatory diseases were excluded from COVID-19 vaccine trials [11, 12]. Therefore, an optimal vaccine strategy for patients with RA receiving MTX is urgently needed.

After vaccination, the following steps of immune responses occur to generate antibodies against vaccine antigens [13]. The vaccine antigens/adjuvants activate dendritic cells at the injection site and induce their migration to draining lymph nodes. In response to vaccine antigens reaching the lymph nodes via antigen-bearing dendritic cells, B cells capable of binding to the antigen with their surface immunoglobulins undergo brisk activation. In an extrafollicular reaction, B cells rapidly differentiate in the plasma cells that produce low-affinity antibodies that appear at low levels in the serum within a few days after vaccination. Antigen-specific helper T cells that have been activated by antigen-bearing dendritic cells trigger some antigen-specific B cells to migrate toward follicular dendritic cells, initiating germinal center (GC) reaction. In GCs, B cells receive additional signals from follicular T cells (Tfh) and undergo massive clonal proliferation; switch from IgM to IgG, IgA, or IgE; undergo affinity maturation; and differentiate into short-lived plasma cells that secrete large amounts of antigen-specific antibodies. At the end of the GC reaction, a few plasma cells (long-lived plasma cells) exit the lymph nodes and migrate to survival niches primarily located in the bone marrow. Memory B cells are generated in response to vaccine antigens during the GC reaction in parallel to plasma cells. Memory B cells transiently migrate through the blood toward the extrafollicular areas of the spleen and lymph nodes. On booster vaccination, memory B cells readily proliferate and differentiate into plasma cells that secrete large amounts of high-affinity antibodies that can be detected in the serum within a few days after boosting.

There is a possibility to infer the stage at which immune response is impaired in patients taking MTX by observing changes in antibody titers over time in individual cases. This information is essential for optimizing the COVID-19 vaccine strategy in patients with RA taking MTX. This study describes the time course of antispike (S) antibody (Roche Elecsys Anti-SARS-CoV-2 S) titers after vaccination with BNT162b2 mRNA COVID-19 vaccine (BioNTech/Pfizer) in eight healthcare workers (HCWs). To the best of our knowledge, this study is the first to examine changes in anti-spike antibody levels over time from immediately after primary vaccination to after booster vaccination in a patient with rheumatoid arthritis taking MTX following BNT162b2 vaccination.

## 2. Case Presentation

Among the eight HCWs who were vaccinated with the BNT162b2 mRNA COVID-19 vaccine, one HCW was diagnosed with RA and was taking MTX. The HCW with RA (patient with RA) was a 60-year-old Japanese male who had been diagnosed with seropositive RA since 2010. The remaining seven HCWs were aged 40–58 years (median 49 years), and there were three females and four males. Details of the characteristics of the participants in this study are presented in Table 1. Obesity and comorbidities were observed in older HCWs. Although age and smoking have been reported as risk factors for lower antibody titers after COVID-19 vaccination [4, 9, 14, 15], no clear association was observed between antibody titers and age or smoking, possibly owing to the small number of participants (Table 1, Supplementary Table S1).

The patient with RA was in remission after MTX (8 mg/week) and bucillamine (200 mg/day) treatment. MTX was withdrawn for 1 week after each vaccination according to the American College of Rheumatology (ACR) guidance [16]. All participants received a primary vaccination of two doses (30 *μ*g each) in April and May 2021 (3-week interval) and a booster vaccination (single dose, 30 *μ*g) in January 2022. Anti-S antibody titers were measured at 10 days, 7 months, and 8 months (1-month postbooster) after the second vaccination. In the patient with RA and in a poor responder HCW whose anti-S antibody titers at 10 days did not reach the upper detection limit, anti-S antibody titers were also measured at 4 months. The upper detection limit of the anti-S antibody titer was set to 999 U/mL at 10 days and 4 months and 9,999 U/mL afterward.

**Figure 1:**
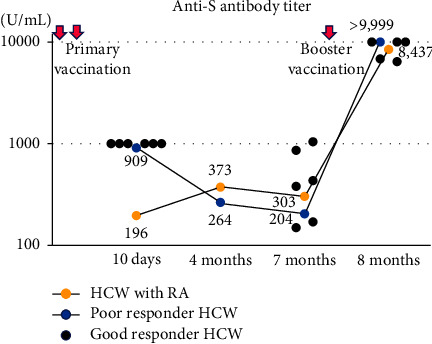
Anti-S antibody titer after primary vaccination and before and after booster vaccination. Anti-S antibody titer levels are shown for the RA patient taking MTX (yellow circle), poor responder HCW (blue circle), and good responder HCW (black circle). The time course of the titers of the RA patient and poor HCW are illustrated by solid lines. The measured values of good responder HCWs are presented in Supplementary Table S1. Overlapping circles are displayed as horizontal shifts. Anti-S antibody: antispike antibody, RA: rheumatoid arthritis. HCW: healthcare worker, MTX: methotrexate.

The anti-S antibody titers of seven HCWs indicated at 10 days were beyond the upper limit of the assay in six (good responders) of seven HCWs and 909 U/mL in one (poor responder) (Figure 1). In the patient with RA, the titer was significantly lower (196 U/mL). At 4 months, the titer of the patient with RA increased to 373 U/mL (1.9-fold increase), which was higher than that of the low-responder HCW (264 U/mL) and similar to the median titer of 380 U/mL in the other HCWs at 7 months (Supplementary Table S1). One-month postbooster, the titer of the patient with RA was 8,437 U/mL, while four HCWs had titers above the upper limit. The titers for the other two HCWs were 6,415 U/mL and 6,817 U/mL, while data were unavailable for one HCW.

## 3. Discussion

The anti-S antibody titer 10 days after primary vaccination (two doses, a 3-week interval) was significantly suppressed in the patient with RA compared to that in poor and good responders of HCWs. Specifically, the anti-S antibody titer was suppressed by one-fifth in the poor responder (196 U/mL vs 909 U/mL). Similarly, there are several reports of the suppression of anti-S antibody titer after primary vaccination with the BNT162b2 mRNA COVID-19 vaccine in patients taking MTX. Bugatti et al. reported that the production of anti-S antibody on the 21st day after a single dose of BNT162b2 mRNA COVID-19 vaccine in patients with chronic inflammatory arthritis taking MTX was significantly lower than that in those who did not take MTX [3]. Haberman et al. reported that patients with immune-mediated inflammatory diseases (IMIDs) on background MTX achieved anti-S antibodies production in only 62.2% of cases, whereas healthy subjects and patients with IMIDs on biologic treatments demonstrated robust antibody responses (over 90%) after primary vaccination (two doses, a 3-week interval) with the BNT162b2 mRNA COVID-19 vaccine [5]. Lukaszuk et al. monitored anti-S antibody titers in a female patient with RA taking MTX and in 119 HCWs for 180 days after the second dose of the BNT162b2 mRNA COVID-19 vaccine using the same assay kit (Roche Elecsys Anti-SARS-CoV-2 S) that we used [7]. In this study, on the 8th day after the second vaccination dose, 68.1% of HCWs had results above 2500 U/mL, which is the upper detection limit of the assay set by Lukaszuk et al. The participants whose results were detected within the upper limit had an average antibody titer of 1670.6 U/mL on day 8, 1319.0 U/mL (21% decrease) on day 14, and 845 U/mL (35% decrease) on day 30 after the second dose. The patient with RA taking MTX had anti-S antibody titers of 0.595 U/mL on day 8, 2.87 U/mL on day 14, and 125.8 U/mL on day 30 after the second vaccine dose. These results may suggest that robust anti-S antibody production by short-lived plasma cells immediately after the second vaccination was strongly suppressed in patients taking MTX. MTX-treated patients may not have had sufficient protection against SARS-CoV-2 infection during the early priming phase. In fact, a higher rate of breakthrough infection has been reported in patients with RA during the priming phase [17].

Lukaszuk et al. continued to monitor anti-S antibody titers and reported that anti-S antibody titers gradually increased to a maximum of 3 months (203,7 U/mL), were maintained for up to 4 months (201.0 U/mL), and decreased slightly at 6 months (147.2 U/mL) after the second vaccination in patient with RA taking MTX [7]. In contrast, in HCWs, the anti-S antibody titers continued to decrease after the second vaccination. Coppeta et al. also reported that anti-S antibody titers in HCWs decreased during the 9 months after primary vaccination (two doses, a 3-week interval) [18]. Therefore, in the late priming phase, anti-S antibody titers increased in patients with RA taking MTX, while they decreased in HCWs. This unique change in anti-S antibody titers in patients with RA taking MTX may suggest that long-lived plasma cells efficiently produced the anti-S antibody even under MTX treatment in the late priming phase.

In a placebo-controlled, observer-blinded, multinational efficacy trial, the efficacy of BNT162b2 was reported to be 96.2% at 2 months, 90.1% at 4 months, and 83.7% at 6 months, with an average decline of approximately 6% every 2 months [12]. The effective rate at 6 months was still considerably high. The anti-S antibody titer at 4 months in the patient with RA in the present study was higher than that in the low responder HCW and similar to the median titer in the other HCWs at 7 months. Therefore, the patient with RA taking MTX may have acquired protection against COVID-19 at 4 months after the second vaccination.

The anti-S antibody titer immediately after the second vaccination was 196 U/mL in our case and even lower at 0.595 U/mL in Lukaszuk's case. This difference may be due to two factors: the MTX dose and the presence or absence of MTX withdrawal after vaccination. The MTX dose in our case was 8 mg/week compared with 25 mg/week in Lukaszuk's case. In our case, MTX was withdrawn for 1 week after vaccination according to the ACR guidance [16] but not in Lukaszuk's case.

One-month postbooster, the anti-S antibody titer of the patient with RA taking MTX drastically increased to a level similar to that of HCWs. Alback et al. reported that anti-S antibody titers in a RA patient receiving MTX and upadacitinib, who received four doses of BNT162b2 irregularly, did not increase after priming vaccination with two doses of BNT 162b at a 3-week interval [2]. Five weeks after the second dose, the patient received a booster vaccination of two doses of BNT162b2 at 3-week interval. The anti-S antibody titers began to increase after the third dose and increased rapidly after the fourth dose. Although there were some significant differences between Alback's and our case, such as the interval and frequency of vaccination, the method of measuring anti-S antibodies, and the absence or presence of MTX withdrawal, they were consistent in that anti-S antibody titers were markedly elevated in patients with RA receiving MTX after booster vaccination. After booster vaccination, antigen-specific antibodies are produced by plasma cells that differentiate from the memory B cells. Therefore, memory B cells specific to the spike antigen must be generated efficiently in the GC during the priming phase and may contribute to vaccination efficacy against severe COVID-19 in patients with RA receiving MTX via the anti-S antibody response immediately after SARS-CoV-2 infection.

In patients with RA, MTX is used as an anchor drug in combination with biologics and JAK inhibitors. It is difficult to examine the effects of individual drugs [4, 6, 9, 10]. In contrast, in patients with psoriasis, MTX or biologics are administered as monotherapy, without steroids. Mahil et al. focused on this point and reported anti-S antibody levels 28 days after a single dose of BNT162b2 in psoriasis patients treated with MTX or biologic monotherapy [19]. MTX has been associated with lower seroconversion rates than either healthy controls or targeted biological therapies. Therefore, suppression of anti-S antibody production in the early priming phase seems to be a phenomenon peculiar to MTX.

Mahil et al. also investigated the cellular immune responses after a single dose of BNT162b2. Peripheral blood mononuclear cells (PBMCs) were stimulated with two separate SARS-CoV-2 spike protein peptide pools. IFN*γ*, IL-2, IL-17A/IL-22 responses by T cells were detected by a direct FluoroSpot assay, and the IL-21 response by Tfh was detected by a direct ELISPOT assay. The production of these cytokines by T cells was not attenuated in patients receiving MTX compared to controls or patients receiving targeted biologics. Since Tfh-derived IL-21 is crucial for memory B-cell responses [20], preservation of IL-21 production may enable memory B-cell generation in patients with RA taking MTX.

Although MTX is one of the first examples of intelligent drug design, multiple mechanisms potentially contribute to its anti-inflammatory actions of MTX, including the inhibition of purine and pyrimidine synthesis, transmethylation reactions, translocation of nuclear factor-*κ*B (NF-*κ*B) to the nucleus, signaling via the Janus kinase (JAK)–signal transducer and activator of transcription (STAT) pathway, nitric oxide production, and the promotion of adenosine release and expression of certain long noncoding RNAs [21]. By modulating cell-specific signaling pathways, MTX inhibits the important proinflammatory properties of major cell lineages involved in RA pathogenesis, including B cells, T cells, macrophages, endothelial cells, and fibroblast-like synoviocytes. Elucidating the mechanism of MTX on the immunogenicity of the COVID-19 vaccine would contribute to the development of optimal vaccination regimens for patients taking MTX, as well as the optimization of MTX treatment itself.

In conclusion, the patient with RA taking MTX showed efficient anti-S antibody production in the late priming phase and after booster vaccination. However, anti-S antibody titers did not increase sufficiently during the early priming phase. The findings of this report may suggest that in patients with RA taking MTX, the generation of short-lived plasma cells was strongly suppressed; in contrast, the generation of long-lived plasma cells and memory B cells was intact. For MTX-treated patients with RA, it is important to complete the primary and booster vaccination series to ensure sufficient immunity against SARS-CoV-2. As our report presents only one case, further investigations using larger samples are required.

## Figures and Tables

**Table 1:** Demographic characteristics of the study population.

	Age (years)	Gender	Smoking	Body Mass index (kg/m^2^)	Disease
RA patient	60	Male	+	21.0	Rheumatoid arthritis
Low responder	42	Female	+	17.2	
Good responder 1	40	Male	−	20.3	
Good responder 2	45	Female	N/A	N/A	
Good responder 3	49	Male	−	21.1	Sleep apnea syndrome
Good responder 4	49	Female	−	24.9	Hyperlipidemia
Good responder 5	51	Male	+	31.6	Diabetes mellitus
Good responder 6	58	Male	+	25.4	Hypertension

## Data Availability

All data of this study are presented in the text, in the table, in the figure or in the supplementary table.

## Supplementary Materials

Supplementary Materials Supplementary Table S1 presents anti-S antibody titer after primary vaccination as well as before and after booster vaccination.
