# Supplementary material for: Time Course of Antispike Antibody Titer after Administration of BNT162b2 mRNA COVID-19 Vaccine in a Patient with Rheumatoid Arthritis on Methotrexate
Source: Case Rep Rheumatol. 2023 Apr 19;2023:4525249. doi: 10.1155/2023/4525249 (PMC10132894; doi:10.1155/2023/4525249)
Supplement: Supplementary Materials — Supplementary Table S1 presents anti-S antibody titer after primary vaccination as well as before and after booster vaccination. [file 4525249.f1.docx]

Supplementary Table S1. Anti-S antibody titer after primary vaccination as well as before and after booster vaccination
